# Network pharmacology-based strategy to investigate the active ingredients and molecular mechanisms of *Scutellaria Barbata D. Don* against radiation pneumonitis

**DOI:** 10.1097/MD.0000000000027957

**Published:** 2021-11-24

**Authors:** Ping-Yi Sun, Ai-Shuai Wang, Zhen-Fei Zhang, Yan-Li Zhang, Xin Zheng

**Affiliations:** a Shandong University of Traditional Chinese Medicine, Jinan 250000, China; b Department of Traditional Chinese Medicine, The First Affiliated Hospital of Shandong First Medical University, Jinan 250000, China; c Heze Hospital of traditional Chinese Medicine, Heze 274000, China; d Qingdao Hospital of Traditional Chinese Medicine (Qingdao Hiser hospital), Qingdao 266000, China.

**Keywords:** mechanism of action, network pharmacology, radiation pneumonitis, *Scutellaria Barbata D. Don*

## Abstract

**Introduction:**

Herbal medicines combined with radiotherapy significantly reduced the incidence of radiation pneumonitis (RP), and the *Scutellaria barbata D. Don (SBD)* is a perennial herb that has been reported to protect against radiation-induced pneumonitis. However, the exact molecular mechanism is not known. The objective of this research was to investigate the against radiation pneumonitis ingredients and their functional mechanisms in *SBD*.

**Methods:**

Based on the network pharmacology approaches, we collected active ingredients and target genes in *SBD* against RP through Traditional Chinese Medicine System Pharmacology (TCMSP) Database, and the “Herb–Ingredients–Target Genes–Disease” Network was constructed by using of Cytoscape. STRING analysis was performed to reveal the protein-protein interactions, and then we applied enrichment analysis on these target proteins, gene function, and pathways.

**Results:**

A total of 18 ingredients in *SBD* regulate 65 RP related target proteins, which show that quercetin, luteolin, baicalein, wogonin may be the key active ingredients, while IL6, AKT1, VEGFA, MMP9, CCL2, prostaglandin-endoperoxide synthase 2 (PTGS2) (cyclooxygenase-2 [COX-2]), CXCL8, IL1B, mitogen-activated protein kinase (MAPK1), and IL10 were identified as critical targets. Besides, the results of Gene Ontology (GO) enrichment analysis and Kyoto Encyclopedia of Genes and Genomes (KEGG) pathway enrichment analysis indicated that predicted targets of *SBD* are mostly associated with the pathological process of oxidative stress and inflammation. AGE- Receptor of Advanced Glycation Endproducts (RAGE) signaling pathway in diabetic complications, IL-17 signaling pathway, hypoxia-inducible factor-1 (HIF-1) signaling pathway, NF-kappa B signaling pathway might serve as the principal pathways for RP treatment.

**Conclusion:**

In our study, the pharmacological and molecular mechanism of *SBD* against RP was predicted from a holistic perspective, and the results provided theoretical guidance for researchers to explore the mechanism in further research.

## 1 Introduction

Radiation therapy may be curative in many types of cancer and also be used as part of adjuvant therapy to prevent tumor recurrence after surgery to remove a primary malignant tumor. However, the lung is more sensitive to the effects than other organs, and radiation pneumonitis is the significant side effect of thoracic radiation therapy^[1]^ that can occur leading to pulmonary insufficiency and death. As the meta-analysis showed that herbal medicines combined with radiotherapy significantly reduced the incidence of radiation pneumonitis.^[2]^ Traditional Chinese Medicine (TCM) is widely used in China for thousands of years,^[3]^ and also practiced outside of China in later years. It is characterized by “multiple ingredients,” “multiple targets,” and “multi-pathway” in disease treatment.^[4,5]^ Based on the traditional Chinese and Korean medicine theory, radiation therapy is regarded as a heat toxin pathogen.^[2]^

*Scutellaria barbata D. Don (SBD)* is a perennial herb which is known in TCM as *Ban-Zhi-Lian*, and the efficacy is mainly in heat-clearing and detoxifying properties (Qingre Jiedu in Chinese).^[6]^*SBD* extraction and functional components were shown to possess vital biological activities like “antioxidant activity,” “reducing apoptosis and oxidative stress,” “anti-inflammatory activity,”^[7–9]^ furthermore, some components have been reported to protect against radiation-induced pneumonitis and enteritis.^[10–12]^ However, the pharmacological mechanisms have not yet been clearly explored.

The TCM network pharmacology that integrates the systems biology and in silico technologies provides a systematic research strategy that conforms to the systematic and holistic perspective of the TCM theory,^[13,14]^ has been widely applied to elucidate the function mechanisms for various diseases, such as cancer, cardiovascular disease, and metabolic disease.^[15–21]^ In this research, we used the network pharmacology approach to systematically explored the mechanism of *SBD* in RP treatment by analyzing the active ingredients, potential target genes, and critical pathways.

## 2 Methods

### 2.1 Screening of active ingredients and target genes for *SBD*

All ingredients of *SBD*, as well as the small molecular structure information of the active ingredients, were collected from the Traditional Chinese Medicine System Pharmacology (TCMSP) Database^[22]^ (http://tcmspw.com/tcmsp.php 2020.03.16). The database is a unique systems pharmacology platform of Chinese herbal medicines that captures the relationships between drugs, targets, and diseases. It collected all the 499 herbs registered in Chinese pharmacopeia (2010), with a total of 12144 chemicals.

“Oral bioavailability” (OB) is an essential criterion for evaluating the quality of medicines. It reflects the proportion of the dose about an orally administered drug that enters the systemic circulation. “Drug likeness” (DL) refers to the similarity of a compound to a known drug. “Drug-like” compounds are not drugs, but they have the potential to become drugs. In this study, the 2 crucial absorption, distribution, metabolism, excretion-related pharmacokinetics parameters were used to screen the active ingredients of *SBD*. As recommended in several articles,^[23–25]^ the ingredients with OB ≥30% and DL ≥0.18 are considered to have better pharmacologic effects and are selected as candidate ingredients for the next step. According to the literature, scutellarin (OB 2.64% and DL 0.79) has been researched and proved to be one of the primary ingredients in *SBD*,^[6,26,27]^ so we incorporated it into our study for further analysis.

All the protein targets of bioactive ingredients in *SBD* were collected from TCMSP Then we use the UniProt knowledge database (https://www.uniprot.org/) with the selected species as Homo sapiens to transform the targets and obtain gene symbols.^[24]^

### 2.2 Acquisition of target genes for radiation pneumonitis

The target genes of radiation pneumonitis (RP) were obtained from the following 2 databases. Genecards (https://www.genecards.org/,2020.03.16) database provides genomic, proteomic, transcriptomic, genetic, and functional information on all known and predicted human genes.^[28,29]^ The Online Mendelian Inheritance in Man^[30]^ (Online Mendelian Inheritance in Man https://omim.org/,2020.03.16) is a continuously updated catalog of human genes and genetic disorders and traits, with a particular focus on the gene-phenotype relationship. We set the keyword as “Radiation pneumonia” and then retrieved the detailed information of the target genes for RP.

### 2.3 Construction of herb–ingredients–target genes–disease network

To illustrate the interactions among herb (*SBD*), ingredients, target genes, and disease (RP), a network of complex information was generated by Cytoscape 3.7.2 (https://cytoscape.org/,2020.03.17)^[31]^: an open-source bioinformatics software platform for visualizing molecular interaction networks and integrating with gene expression profiles and other state data. In the network plot, nodes represent the RP /*SBD*/ingredients/ target genes, while edges stand for that they are linked with each other.

### 2.4 Protein-protein interaction network

STRING (Search Tool for the Retrieval of Interacting Genes/Proteins https://string-db.org/, 2020.03.17) is a biological database and web resource of known and predicted protein-protein interactions.^[32,33]^ In STRING, we set the common target proteins with species as “Homo sapiens” and confidence score >0.4, then exported the PPI results as an image. The network nodes represent proteins, while the edges represent protein-protein associations.

### 2.5 Enrichment analysis

GO^[34]^ is an international standard classification system for gene function. It aims to establish a language vocabulary standard that is suitable for various species, defines and describes the functions of genes and proteins, and can be updated as research continues. GO enrichment analysis interprets the biological function of target genes in terms of gene function. In this study, we use the R Project for Statistical Computing (R 3.6.3 https://www.r-project.org/) to perform it.

KEGG is an effort to link a set of genes in the genome with a network of interacting molecules in the cell, such as a pathway or a complex, representing a higher-order biological function.^[35]^ The KEGG pathway enrichment analysis of overlapping target genes was executed by the R Project for Statistical Computing (R 3.6.3 https://www.r-project.org/).

## 3 Results

### 3.1 Active ingredients and target prediction

**Figure 1:**
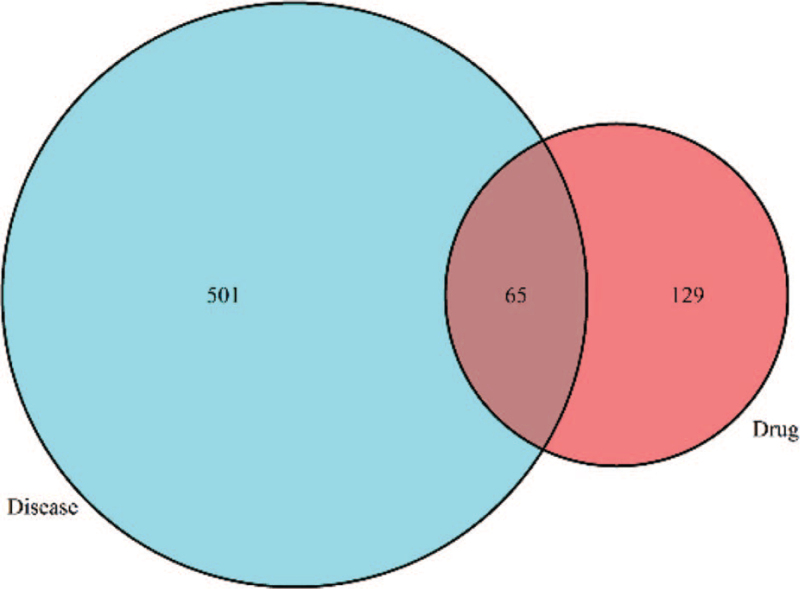
Sixty five overlapping targets in *Scutellaria Barbata D. Don* against Radiation Pneumonitis. The figure constructed using the R Project for Statistical Computing (https://www.r-project.org/).

After the elimination of redundant items, 30 active ingredients were selected from the *SBD* (Table 1  ), and 194 known target symbols related to the ingredients were obtained (Supplementary material file S1) by the TCMSP database. A total of 566 known therapeutic target genes for RP were collected (supplementary material file S2). Then we acquired 65 overlapping target genes for *SBD* and RP (Fig. 1), and the gene symbols are listed in the Supplementary material file S3.

**Table 1:**
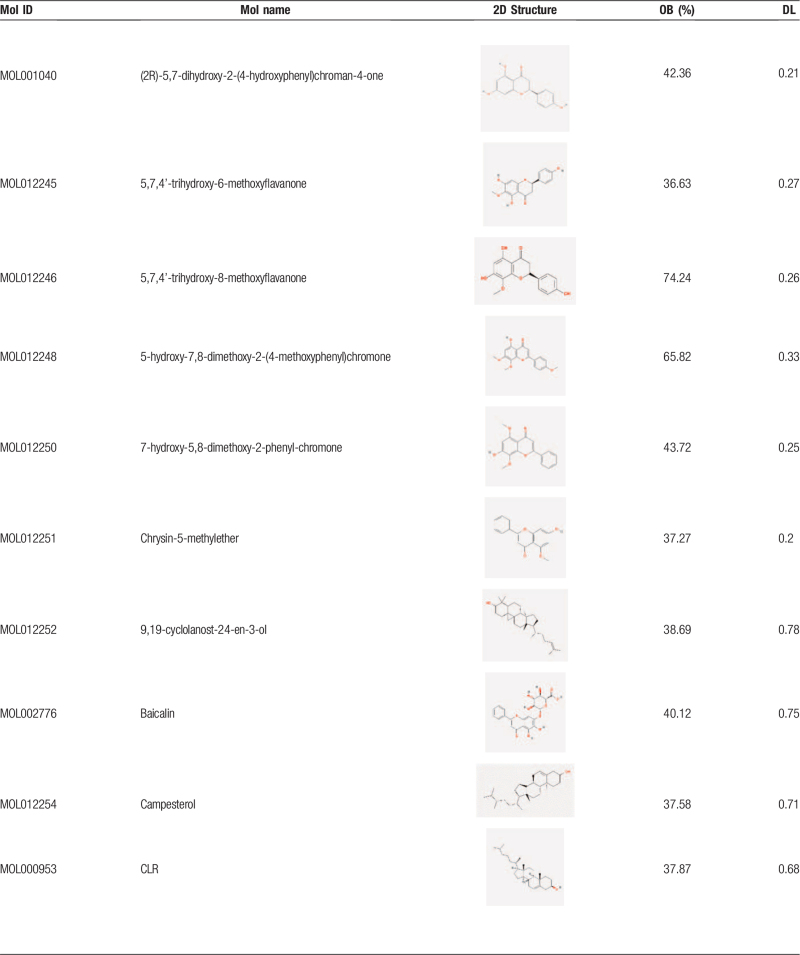
Active ingredients of *Scutellaria Barbata D. Don*. (Put behind paragraph 3.1).

**Table 1 (Continued):**
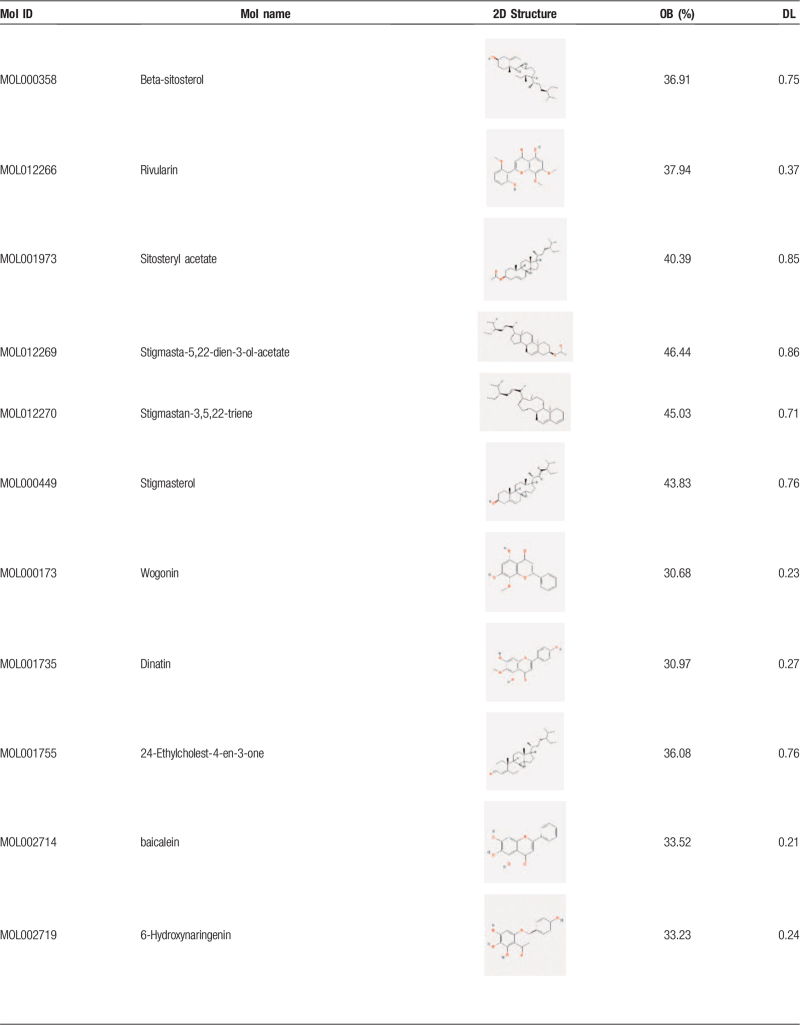
Active ingredients of *Scutellaria Barbata D. Don*. (Put behind paragraph 3.1).

**Table 1 (Continued):**
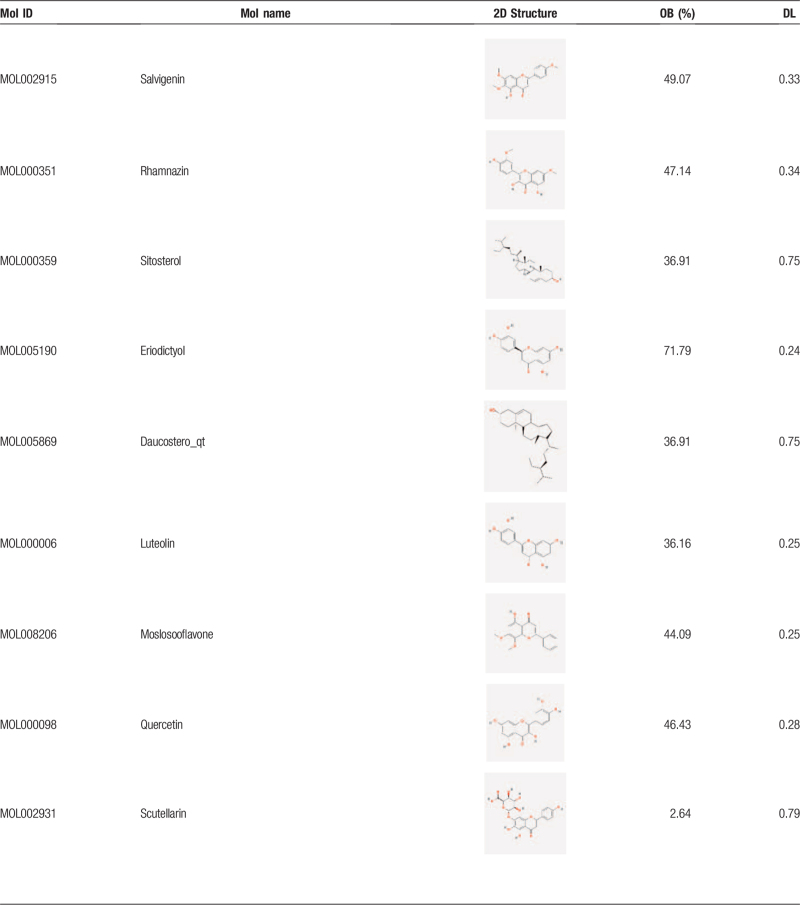
Active ingredients of *Scutellaria Barbata D. Don*. (Put behind paragraph 3.1).

### 3.2 Herb–ingredients–target genes–disease network analysis

**Figure 2:**
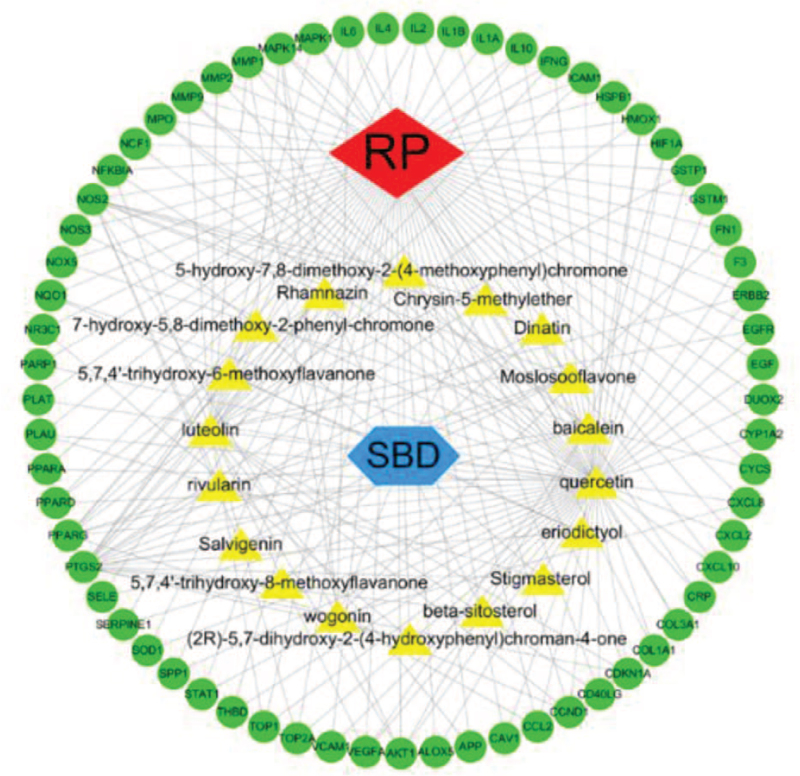
H–I–T–D network generated by Cytoscape 3.7.2 (https://cytoscape.org/). The red diamond node represents RP, and the blue hexagon node represents *SBD*. The 18 yellow triangle nodes represent the active ingredients in *SBD*; The 65 green ellipse nodes represent the overlapping gene symbols between the disease and drug. The edges denote that nodes can interact with each other. RP = Radiation pneumonitis.

**Figure 3:**
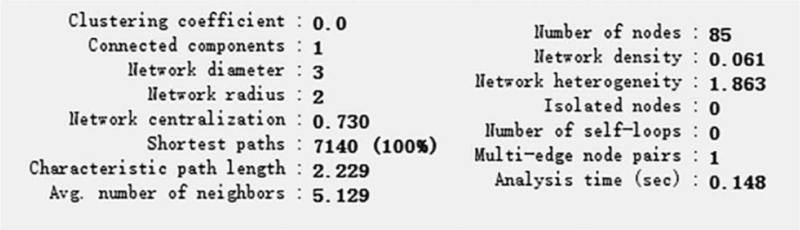
The detailed parameters of H–I–T–D network. H–I–T–D = Herb–Ingredients–Target Genes–Disease.

**Figure 4:**
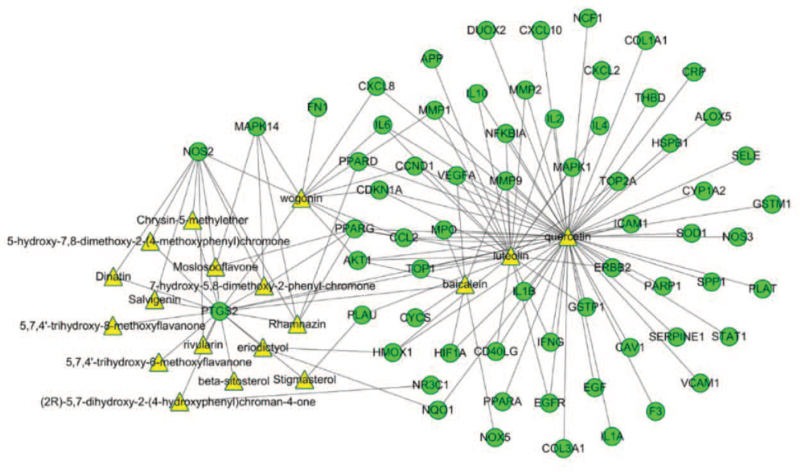
Ingredient-Target network created by Cytoscape 3.7.2 (https://cytoscape.org/). The yellow triangle represents the active ingredients in SBD, the green ellipse represents the targets, and the edge represents the correlation between them.

Herb–ingredients–target genes–disease network illustrates the interrelationship for the ingredients and related protein targets in *SBD*/RP (Fig. 2), a total of 65 target genes associated with 18 active ingredients were selected. We can see that some targets were hit by multiple ingredients, while 1 ingredient can act on multiple targets. The detailed parameters are shown in Figure 3. In this network, the red diamond node represents RP, and the blue hexagon node represents *SBD*. While the 18 yellow triangle nodes represent the selected active ingredients in *SBD* that may act against RP, and the 65 green ellipse nodes represent the common target genes. The edges indicate that nodes can interact with each other. Figure 4 shows the ingredient-target network diagram, the yellow triangle represents the active ingredients of *SBD*, the green ellipse represents the targets, and the edge represents the correlation between them.

### 3.3 Protein-protein interaction network and key targets prediction

**Figure 5:**
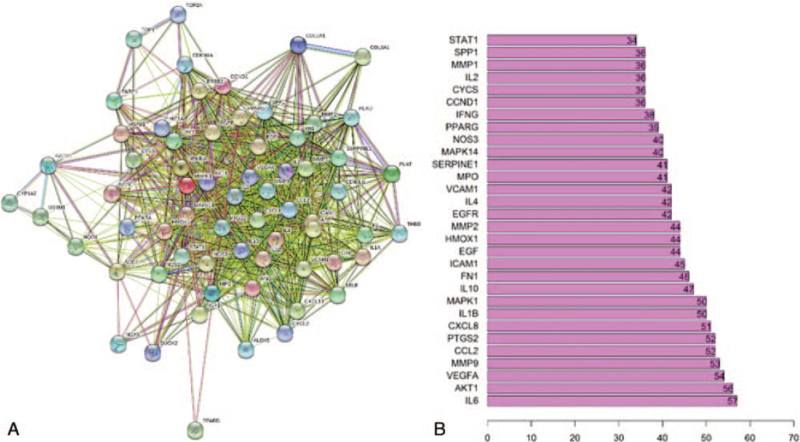
A: The PPI network exported from STRING (https://string-db.org/) database. B: The barplot of the first 30 proteins in the PPI network. The x-axis represents the number of neighboring proteins of the target protein. The y-axis represents the target protein.

We imported the 65 common target genes into the STRING database to generate the PPI network (Fig. 5A). The light-blue edges represent known interactions from curated databases. The pink edges represent the known interactions experimentally determined. The green, red, dark-blue edges represent that the predicted interactions arose from neighborhood gene, gene fusions, and gene co-occurrence, respectively. While the yellow, black, and lavender edges represents arousing from text mining, co-expression, and protein homology, respectively. (https://string-db.org/cgi/network.pl?taskId=Iy6OMVaVsrs3&sessionId=S8OorXbxYZAu&bottom_page_content=table).

Then we took the first 30 proteins in the network. As seen in Figure 5B, the focus of our research of PPIs probably were IL6, AKT1, VEGFA, MMP9,CCL2, prostaglandin-endoperoxide synthase 2 (PTGS2), CXCL8, IL1B, mitogen-activated protein kinase (MAPK1), IL10, FN1, intercellular cell adhesion molecule-1, EGF, HMOX1, MMP2, EGFR, IL4, vascular cell adhesion molecule 1, MPO, etc. The results suggested that these proteins including IL6 (count = 57), AKT1 (count = 56), VEGFA (count = 54), MMP9 (count = 53), CCL2 (count = 52), PTGS2 (count = 52), CXCL8 (count = 51), IL1B (count = 50), MAPK1 (count = 50), IL10 (count = 47) would be the key targets for *SBD* acting against RP.

### 3.4 Gene ontology enrichment analysis

**Figure 6:**
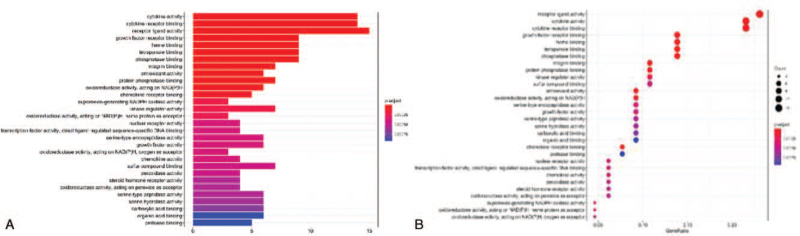
GO analyses executed by the R Project for Statistical Computing (https://www.r-project.org/). A: Barplot; B: Dotplot. The x-axis represents significant enrichment in the counts or GeneRatio of these terms. The y-axis represents the categories of “biological process” in the GO of the target genes (*P* < .01).

In order to elucidate the relevant biological function, we conducted GO enrichment analysis. (*P* < .01) (Supplementary material file S4) The smaller the value of p. adjust, the greater correlation and importance. The x-axis represents the number of enriched genes (Barplot) and the ratio of the gene (Dotplot), while the y-axis represents GO terms. As the result shown, numerous biological processes associated with the research mechanism, then we intercepted the top 30 terms based on the *P* value (Fig. 6A and B), including cytokine activity (GO:0005125); cytokine receptor binding (GO:0005126); receptor ligand activity (GO:0048018); growth factor receptor binding (GO:0070851); heme binding (GO:0020037); tetrapyrrole binding (GO:0046906);phosphatase binding (GO:0019902); integrin binding (GO:0005178); antioxidant activity (GO:0016209); protein phosphatase binding (GO:0019903); oxidoreductase activity, acting on NAD(P) H (GO:0016651); chemokine receptor binding (GO:0042379); kinase regulator activity (GO:0019207), etc.

### 3.5 Kyoto encyclopedia of genes and genomes pathway enrichment analysis

**Figure 7:**
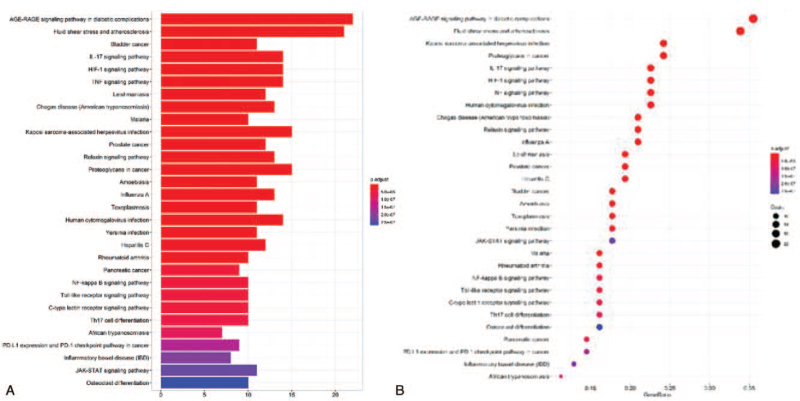
KEGG pathway enrichment analyses performed by the R Project for Statistical Computing (https://www.r-project.org/). A: Barplot; B: Dotplot. The x-axis represents the counts or ratio of the target symbols in each pathway, and the y-axis represents the main pathways (*P* < .01).

We performed KEGG enrichment analysis (*P* < .01) (Supplementary material file S5) on the common targets shared by the *SBD* ingredient targets and RP-related targets, of which the first 30 enriched are presented in Figure 7A and B. The smaller the value of p. adjust, the more significant correlation and importance. The x-axis represents the number of genes (Barplot) or the ratio of the gene (Dot plot) enriched in the pathway, and the y-axis represents the KEGG pathway. This result indicated that the key pathways responsible for RP treatment might focus on the coordinated regulation of several inflammation-related pathways, including AGE- Receptor of Advanced Glycation Endproducts (RAGE) signaling pathway in diabetic complications(hsa04933), IL-17 signaling pathway (hsa04657), hypoxia-inducible factor-1 (HIF-1) signaling pathway (hsa04066), tumor necrosis factor (TNF) signaling pathway (hsa04668), NF-kappa B signaling pathway (hsa04064), Toll-like receptor signaling pathway (hsa04620), JAK-STAT signaling pathway (hsa04630).

## 4 Discussion

Histopathologically, radiation pneumopathy is characterized by “loss of epithelial cells; edema; inflammation; occlusions airways; air sacs and blood vessels; fibrosis tissue injury”.^[36]^ The primary mechanisms of tissue injury include direct DNA damage and the generation of reactive oxygen species (ROS),^[37]^ while cellular injury leads to inflammatory cell infiltration and cytokine release.^[36,38]^ After radiation, the injured cells lead to the release of chemoattractant molecules, which can stimulate neutrophils arriving into the irradiated lung, and then promote local inflammation through the release of IL-1, IL-6, and reactive oxygen species.^[39,40]^ As previous researches reports, radical-induced oxidative stress following thoracic irradiation participate in RP,^[41,42]^ and the COX-2 is considered to be a pro-inflammatory enzyme during the oxidative stress.^[43]^

In the present study, according to the PPI network, some important targets exhibit the therapeutic effects, which mainly concentrated in cytokine and protein kinase like IL6, AKT1, CCL2, PTGS2, COX-2, CXCL8, IL1B, MAPK1, IL10. GO and KEGG enrichment analysis together display that the potential key targets participating functions and pathways reflected in this aspects: inflammation; immunity; antioxidant; cell proliferation, differentiation; and apoptosis. In combination with analyses of the constructed network and enrichment results, we anticipate that the pharmacologic mechanism of *SBD* against RP is closely related to oxidative stress and inflammation.

As shown in Figure 6A and B, the same target protein such as IL-1 exists in multiple pathways like the IL-17 signaling pathway, TNF signaling pathway. Simultaneously, there are multiple target proteins involved in 1 pathway like AGE-RAGE signaling pathway in diabetic complications (hsa04933, count = 22), IL-17 signaling pathway (hsa04657, count = 14), HIF-1 signaling pathway (hsa04066, count = 14), TNF signaling pathway (hsa04668, count = 14), NF-kappa B signaling pathway (hsa04064, count = 10). The AGE-RAGE signaling pathway via phosphatidylinositol- 3 kinases, p21-Ras and the MAPKs, extracellular signal-regulated kinase1/2, p38 promote the translocation of nuclear factor-κB (NF-κB) from the cytoplasm to the nucleus, which induce the expression of inflammatory cytokines such as IL6, CCL2, CXCL8, intercellular cell adhesion molecule-1, IL1B, and vascular cell adhesion molecule 1.^[44]^ Interaction of RAGE with advanced glycation end products can also trigger spurs a surge of reactive oxygen species,^[44,45]^ then provoke activation of the p21Ras and MAPKs. In this study, the IL-17 signaling pathway involving IL-17A and IL-17F signals via correspondent receptors to activate downstream pathways that include NF-kappaB, MAPKs and then induce the expression of cytokines and chemokines, which is also shown in the TNF signaling pathway. Besides those, the HIF-1 signaling pathway is noteworthy. HIF-1 acts as a master regulator of numerous hypoxia-inducible genes under hypoxia conditions, while it is induced not only in response to reduced oxygen availability but also by other stimulants, such as nitric oxide, or various growth factors. In reference previous study, the mRNA levels of HIF-1a, as well as of HIF-1 target genes vegfa, cxcl12, pgk1, were found to be up-regulated upon administration of bleomycin and the development of pulmonary inflammation and fibrosis.^[46]^ As shown in the HIF-1 signaling pathway, growth factor can synthesize HIF-1a via phosphoinositide 3-kinase or mitogen-activated protein kinase (MAPK) pathways. Radiation pneumonitis and radiation fibrosis are 2 closely related pathological processes of radiation-induced lung injury. Therefore, the intervention of HIF1 is important for both pneumonitis and later fibrosis.

It is faster and also accurately predict potential active ingredients like quercetin, luteolin, baicalein, wogonin in *SBD* acting on key targets and pathways that may be the key research objects for further experimental studies. Refer to past literature, related studies have been conducted. Baicalein inhibited the expression of NF-κB p65 and the phosphorylation of p38 MAPK, extracellular signal-regulated kinase via dampening the NF-κB and MAPK signaling pathways to exerts its anti-inflammatory effects,^[47]^ and it also inhibited the expression of IL-6, IL-1β, Icam-1, and Vcam-1 in the irradiated intestine.^[12]^ Luteolin suppresses the expression of IL-6, iNOS, COX-2, and ROS by blocking the activation of MAPK and NF-κB pathways in acute lung injury induced by lipopolysaccharide.^[48–50]^ Quercetin liposomes were shown to protect against radiation-induced and Lipopolysaccharide (LPS) induced acute pneumonitis by reducing the MDA content, increasing SOD activity in the lung tissues, and reducing the total cell counts and inflammatory cell proportions in the bronchoalveolar lavage fluid.^[10,51]^ Wogonin reduced LPS-induced neutrophils infiltration, pro-inflammatory cytokines generation, adhesion molecules expression, Akt (PKB) phosphorylation, RhoA activation, and also prevented LPS-induced acute lung injury via regulating the PPARγ-involved NF-κB pathway.^[52,53]^ However, the network pharmacology results and above literature reports partly reflects the effectiveness and mechanism, while the exact key ingredients and pharmacological mechanism of *SBD* acting on RP should be validated by further experimental studies.

## 5 Conclusion

In conclusion, *SBD* is widely used for inflammation and cancer, the pharmacological mechanism of interfering with lung cancer has been described,^[54]^ but RP has not. Our research applied a network approach to display that active ingredients in *SBD* probably play an essential part against RP by participating in the regulation of oxidative stress and inflammation, and the selected key targets, as well as signal pathways, can provide a reference for further experiments to clarify the precise mechanism.

## Author contributions

**Conceptualization:** Xin Zheng.

**Data curation:** Pingyi Sun, Ai-Shuai Wang, Zhen-Fei Zhang, Yan-Li Zhang.

**Methodology:** Pingyi Sun.

**Visualization:** Pingyi Sun.

**Writing – original draft:** Pingyi Sun.

## Supplementary Material

Supplemental Digital Content

## Supplementary Material

Supplemental Digital Content

## Supplementary Material

Supplemental Digital Content

## Supplementary Material

Supplemental Digital Content

## Supplementary Material

Supplemental Digital Content
